# Vaccination hesitancy: To be vaccinated, or not to be vaccinated, that is the question in the era of COVID‐19

**DOI:** 10.1111/phn.13134

**Published:** 2022-09-27

**Authors:** Cecilia Perrone, Elena Fiabane, Marina Maffoni, Antonia Pierobon, Ilaria Setti, Valentina Sommovigo, Paola Gabanelli

**Affiliations:** ^1^ Istituti Clinici Scientifici Maugeri IRCCS Psychology Unit of Pavia Institute Pavia Italy; ^2^ Istituti Clinici Scientifici Maugeri Department of Physical and Rehabilitation Medicine of Nervi Institute Genova Italy; ^3^ Istituti Clinici Scientifici Maugeri IRCCS Psychology Unit of Montescano Montescano Italy; ^4^ Department of Brain and Behavioural Sciences Unit of Applied Psychology University of Pavia Pavia Italy; ^5^ Department of Psychology Faculty of Medicine and Psychology Sapienza University of Rome Roma Italy

**Keywords:** COVID‐19, infodemic, lack of control, vaccination, vaccine hesitancy

## Abstract

COVID‐19 vaccine hesitancy poses serious challenges in achieving adequate vaccine coverage in the general population. While most studies on vaccine hesitance determinants during the COVID‐19 pandemic were quantitative, qualitative research on the reasons for vaccine resistance is still lacking. To fill this gap, this study aims to qualitatively investigate cognitive and emotional factors associated with COVID‐19 vaccine hesitancy. This qualitative pilot study was conducted between October and November 2021 in Italy. A total of 40 COVID‐19 vaccine‐hesitant (“hesitant not vaccinated” or “hesitant but vaccinated”) individuals completed anonymous questionnaires with open‐ended questions. Data were analysed using the Interpretive Description approach. The central theme that emerged about vaccine hesitancy was the lack of control. This construct included four different sub‐categories: distrust of the government, infodemic, influence of family, and general anti‐vaccine opinions. The results also showed that the most important emotional and cognitive factors associated with hesitancy were anger related to a perceived sense of oppression; emotional avoidance to minimize risk; anxiety related to potential vaccine side effects. Identifying and understanding factors influencing COVID‐19 vaccination hesitancy is crucial to improving communication strategies that will ultimately result in increased confidence and vaccine acceptance.

## 1 BACKGROUND

According to data reported by the National Agency for Health Services, in Italy, the number of COVID‐19 and death burden cases corresponds respectively to 11,235,745 and 147,320 (Agenzia Nazionale per i Servizi Sanitari Regionali, 2022). The percentage of people over the age of twelve who received a complete COVID‐19 vaccination course from the start of the pandemic was equal to 77.77% in December 2021. Among Europe countries, Italy has been the fourth most vaccinated country, preceded only by Portugal (87.85%) Malta (84.19%), and Spain (79.71%; Agenzia Nazionale per i Servizi Sanitari Regionali, 2022) .These are reassuring data if we consider that in the recent past Italy was one of the main European countries to record the highest rate of measles cases (Signorelli et al., 2017), thus leaving the complex debate on vaccination open. However, in Italy, vaccination‐uptake levels have been decreasing in the last years, leading to the re‐emergence of infectious diseases, resulting in a call for the national government to introduce compulsory school‐entry vaccination (Bertoncello et al., 2020).

Concerning the current pandemic, the advent of COVID‐19 has inevitably drawn attention to vaccination (Hyland et al., 2021) and its psycho‐social consequences for the general population, prompting the scientific community to address not previously investigated areas.

Since December 27, 2020, the date that marks the beginning of the vaccination campaign in Europe, as well as a symbolic turning point in the fight against COVID‐19 (Alwi et al., 2021; Giuliani et al., 2021), several studies have been published on people's acceptance and attitudes toward COVID‐19 vaccines. Although the research has clarified the main reasons behind an individual's intention to be vaccinated against COVID‐19, to date, too little is still known about the factors that influence an individual's decision to delay or refuse some or all vaccines.

Vaccine hesitancy is the delay in acceptance, reluctance, or refusal of vaccination despite the availability of vaccination services (Sallam, 2021). The World Health Organization (WHO) defines it as “one of the top ten threats to world health,” representing a significant barrier to reaching herd immunity in the population (Sallam, 2021). Vaccine hesitancy exists along a broad continuum ranging from absolute vaccine refusal (i.e., anti‐vaxxers) to mild concern regarding a specific vaccine (i.e., vaccine skeptics). Vaccination hesitancy during this pandemic has previously been investigated mainly in terms of hypothetical questions about the acceptance of the COVID‐19 vaccine when there were still no vaccines developed (Edwards et al., 2021; Fisher et al., 2020; Harapan et al., 2020; Reiter et al., 2020).

More recently, despite the safety and efficacy of COVID‐19 vaccines, people continued to express hesitancy, and vaccine acceptance rates remain low in some countries (Sallam, 2021). For example, Soares et al. (2021) showed that COVID‐19 vaccine hesitancy in Portugal at the beginning of the vaccine campaign was high since 56% wanted to wait and 9% refused it. Many factors can contribute to explaining why vaccine‐hesitant individuals may have skeptical and doubtful attitudes and beliefs toward COVID‐19 vaccination (World Health Organization, 2019). The current literature on COVID‐19 vaccine hesitancy has identified concerns about the rapid pace of vaccine development (Freeman et al., 2020), aversion to side effects (Luyten et al., 2019), and the spread of misinformation about the outbreak (Loomba et al., 2021) as key drivers of vaccine refusal and delay. Additionally, underlying causes of vaccine hesitancy are an intricate interaction between mistrust in government and health authorities (Lazarus et al., 2021) combined with novel misinformation on vaccine safety and disease risk arising daily (Larson & Broniatowski, 2021). Among the other influencing factors, previous studies have identified the following: lower age, loss of income during the pandemic, no intention of taking the flu vaccine, low confidence in the COVID‐19 vaccine and the health service, worse perception of government measures, perception of the information provided as inconsistent and contradictory (Giuliani et al., 2021; Gorman et al., 2021; Soares et al., 2021; Troiano e Nardi, 2021). Additionally, the individual levels of anxiety, fear, and individual risk seem to play an important role in vaccine refusal (Bendau et al., 2021). However, the research has obtained mixed results indicating the need to further investigate which and how emotional states and cognitive‐related factors can affect vaccine hesitancy (Bendau et al., 2021). Although vaccination is an individual choice, vaccine‐hesitant people substantially affect the pandemic trajectory, which may compromise present efforts to contain COVID‐19 with negative implications for the entire healthcare system (Olivera‐Mesa et al., 2022). To date, to the best of our knowledge, while most studies investigating attitudes toward COVID‐19 vaccination among the general population adopted a quantitative approach (Giuliani et al., 2021; Gorman et al., 2021; Larson et al., 2016), only a few empirical investigations have utilized open‐ended questions to reach a deeper understanding of the cognitive and emotional factors characterizing vaccine‐hesitant individuals (e.g., Geana et al., 2021; Morales et al., 2022).To fill this gap, we decided to conduct a qualitative study on a sample of the Italian‐hesitant population, since it is important to deepen the understanding of this phenomenon by exploring the specific beliefs and emotions that influence an individual's decision to delay or refuse the vaccine.

In this context, this qualitative pilot study aimed to investigate cognitive and emotional factors associated with COVID‐19 vaccination hesitancy among a group of Italian hesitant individuals.

## 2 METHODS

This study was conducted between October and November 2021 in Northern Italy. Using the saturation principle (i.e., data collection is unnecessary when theoretical saturation is reached, namely the point at which gathering more data does not provide new information on the topic under investigation), anonymous questionnaires with open‐ended questions were administered online utilizing a spreadsheet on Google Sheets. Using snowball sampling, starting from the authors’ social networks and personal contacts, some research participants were identified who, in turn, recruited more subjects from among their acquaintances. Specifically, participants were asked to provide their opinions on several questions about vaccination hesitancy. The inclusion criteria for this study were the following: a) to be native Italian speakers; b) to be at least 18 years of age; c) to fall into the categories “hesitant not vaccinated” or “hesitant but vaccinated” against COVID‐19. In total, our convenience sample was composed of 40 participants who volunteered to participate in the investigation after providing their informed consent and ensuring the anonymity and confidentiality of their responses. Participants were allowed to submit their responses once only to prevent the collection of multiple responses from the same subject. Based on the existing literature, the survey was developed including questions previously utilized in other studies (Dror et al., 2020; Giuliani et al., 2021) and ad hoc questions specifically developed for this research (see Table S1). To reduce participants’ evaluation apprehension, only a few socio‐demographic characteristics (i.e., age, sex, region of residence) were asked of all participants. Then, the survey included two slightly different sections based on which two statements were selected by the participants as the option that best described them. Participants who chose the first statement, namely “*You are hesitant/doubtful and have NOT yet decided to vaccinate against COVID‐19*,” were clustered as “hesitant not vaccinated.” In contrast, those who selected the second option, namely “*You are hesitant/doubtful but have vaccinated against COVID‐19 out of necessity (e.g., work)*,” were categorized as “hesitant but vaccinated.” The survey section that was addressed to “hesitant not vaccinated” participants investigated the following aspects: 1) the reasons why the participant had not yet received at least one dose of the COVID‐19 vaccine, 2) the personal measures used to deal with the virus, 3) the main information sources from which the participants learned more about vaccines, 4) the most influential source on the choice of not being vaccinated, 5) the emotions that best described the decision of the participant not to be vaccinated, 6) what their main reference figures’ (pro or against) positions were about the vaccination, 7) the determining factors that would lead the participant to choose to be vaccinated, 8) the possible consequences experienced after non‐vaccination. Conversely, the survey section aimed at “hesitant but vaccinated” respondents focused on the following aspects: 1) the main reasons why the participant hesitated and delayed the vaccination, 2) the personal measures used to cope with the virus before the vaccination, 3) the main sources from which they informed themselves about the vaccines, 4) the most influential source on the choice of postponing their vaccination, 5) the emotions that best described their state of indecision and doubt before vaccination, 6) the main emotions the participants experienced after receiving their COVID‐19 vaccinations, 7) what their main reference figures’ (pro or against) positions were about the vaccination, 8) the main factors affecting their decision to be vaccinated, 9) the potential consequences experienced about their previous condition of vaccine hesitation. The study was approved by the Ethics Committee of the University of Pavia – Italy (CEC N° 86/2021). Before participating, each subject was required to sign and read an informed consent, in which the objectives, protocol, and data storage methods were explained, including anonymity and the right to interrupt the fulfilling of the questionnaire at any time without providing explanations.

## 3 DATA ANALYSES

The Interpretive Description approach (i.e., a flexible approach particularly suitable for research in the healthcare sector because it allows deeply investigating new phenomena from the viewpoint of first‐line protagonists, Thorne, 2016), thematic areas describing and explaining the vaccination hesitancy phenomenon were investigated by deeply analyzing the transcripts. Specifically, C.P., P.G., and E.F. read the responses to detect themes and subthemes. The three authors independently performed data coding and then a convergence assessment, discussing any discrepancies in thematic classification. Where no consensus was reached, M.M. made the final decision, performing independently of the data coding. All other authors supervised the analysis and provided critical insights on how to redefine the results during periodic meetings.

## 4 RESULTS

Of the forty vaccine‐hesitant participants, 22 were vaccinated (“hesitant but vaccinated”) and eighteen were unvaccinated (“hesitant not vaccinated”).

Most participants were women (75.0%) with an average age of 41.06 years (SD = 15.59), 53.8% had a university degree, followed by 38.4% with elementary and middle school qualifications and 7.8% had a high school diploma. The majority of the sample lived in Lombardy (45.0%), the Italian region most affected by the pandemic (Percudani et al., 2020).

### 4.1 Vaccination‐hesitancy themes

**FIGURE 1:**
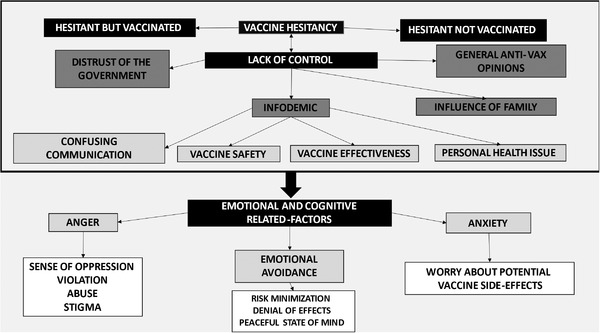
. Conceptualization of the themes. The figure summarizes themes that emerged from the results. “Vaccine hesitancy” was explained in term of perceived “lack of control” which is related to themes of distrust of the government, infodemic, general anti‐vax opinions and influence of family. Infodemic is a complex factor explained by confusing communication, personal beliefs about vaccine safety/effectiveness and personal health issue. Vaccine hesitancy was found to be associated with specific emotional states (anger, emotional avoidance and anxiety) and cognitive‐related factors.

The main theme of hesitancy was the perceived lack of control, which included different subthemes: distrust of the government; infodemic; the influence of family, and general anti‐vax opinions (see Figure 1).

*1) Distrust of the government*

Some participants (25.0%) reported that their perceptions of insecurity were explained by the actions taken by Italian authorities that were described as not reassuring. Individual accountability through signature on the informed consent form and the absence of guarantees or agreements with pharmaceutical companies regarding compensation for any post‐vaccine damages fomented a general distrust and uncertainty toward vaccination.

*2) Infodemic*

The infodemic, or “epidemic of false information” about COVID‐19 refers to a huge amount of unverified information about various aspects of COVID‐19 disease, including disease prevention and control measures, and its consequences, which was spread through social media, television networks, and news agencies (Allahverdipour, 2020). The circulation of an excessive amount of information also about vaccines, sometimes not carefully screened, hindered people's ability to orient themselves. This condition was reflected in the following aspects: confusing communication transmitted by the main sources of information (22.5%), widespread mistrust of the safety (25.0%), and effectiveness (55.0%) of vaccines, fear of developing possible side effects, and concerns about vaccine interference with other medical conditions (25.0%).

*3) Influence of family*

Many participants (50%) cited the influence of their family and/or friends as a factor that contributed to their hesitant position. The majority referred to the presence of at least one family member who supported their scepticism about the vaccine.

*4) General anti‐vax opinions*

Total 12.5% of the sample reported anti‐vaccination opinions related to previous mistrust in the healthcare system, lack of trust in the progress of medicine, and the deep‐seated suspicion of pharmaceutical companies dealing with the general production of vaccines.

The sub‐themes and interviewees’ quotes are reported in Table 1.

**TABLE 1:** Themes and participants’ quotes

**Lack of control**	
**Distrust of the government**	
“If the state signs the consent for me, it would directly expose itself and make me feel safe”; ”I would get vaccinated if there was more sincerity on the part of the institutions”; “I don't feel protected by the state regarding possible side effects of vaccines”	
**Infodemic**	
**Confusing communication**	“I didn't draw from any source for too many conflicting opinions”; “The main sources that influenced the choice of not taking the vaccine are the perceived uncertainty from the television shows and the recurring discordant opinions"; “The main reason for not taking the vaccine is the general confusion created by the media”
**Vaccine validity**	“The reason why I did not get vaccinated is the lack of confidence in the validity and efficacy of a vaccine that is still in its trial phase”; “I don't believe in vaccines”; “The reason why I delayed the vaccination concerns the unproven safety of the vaccine”
**Vaccine security**	“I think that the vaccine can hurt”; “The reason I don't get vaccinated is the fear of short‐ and long‐term side effects”; “I am not sure that there are no consequences”; “I would be vaccinated if I had a guarantee concerning future side effects and vaccine effectiveness over the years”
**Personal health issue**	“I delayed my vaccination because I was scared that it would interfere with my health conditions, especially in the future”; “I would get vaccinated if I received guarantees regarding my current medical records”; “I'm really scared that the vaccine will interfere with other physical problems”; “I'm afraid that my body will react negatively”
**Influence of family**	
“My Family members, who I trust and respect, are against the vaccination”; “My parents do not support the vaccine”; “My father is against it”	
**General anti‐vax opinions**	
“I did not take the vaccine because I think vaccination is unnecessary”; “I am opposed to drugs: moreover, this vaccine seemed to be not fully tested and with unacceptable danger margins”; “I don't get vaccinated because I have already had a negative experience with the flu vaccination”	

### 4.2 Emotional and cognitive related factors

The themes that emerged from hesitancy were associated with specific emotional and cognitive factors: anger, emotional avoidance, and anxiety.

*1) Anger*

Anger was expressed by 30% of respondents as a powerful sense of oppression (“I feel anger and frustration because of the pressures caused by society”), violation (“I feel anxious and violated”), and abuse (“Forcing people to sign a consent is a dictatorship”). While the “hesitant not vaccinated” experienced anger mainly because of experiences of discrimination and social stigma, the “hesitant but vaccinated” reported danger related to the restrictions imposed by the government that forced them to vaccination.

*2) Emotional avoidance*

Emotional avoidance (35% of the total sample) emerged with specific regard for the dimensions of risk minimization (“*I thought I did not need the vaccine given my young age and my optimal state of health*”), denial of effects and consequences (“*I don't consider COVID‐19 as a substantial danger for my lifestyle*”), and expression of positive emotions to counterattack anxiety and worry (“*I feel peaceful and calm*”).

*3) Anxiety*

Anxiety was the most prevalent emotion (57.5%). It refers to concerns and worries about possible side effects of the vaccine. This emotional state was reported by both groups who experienced recurrent negative thinking (“*After the vaccine, I feel anxious, worried and stressed because of the fear of being sick*;” “*I don't get vaccinated because I'm afraid of short‐ and long‐term side effects*”).

The prevalence of both themes and emotional factors is reported in Table 2.

**TABLE 2:** The prevalence of themes, emotional and cognitive factors for the total sample

Lack of control^a^	% (*n*)
Distrust of the government	25.0 (10)
Infodemic	
Confusing information	22.5 (9)
Personal health issue	25.0 (10)
Vaccine safety	25.0 (10)
Vaccine effectiveness	55.0 (22)
Influence of family	50.0 (20)
Generic anti‐vax opinions	12.5 (5)

Emotional factors	
Anger	
Anger (general)	17.5 (7)
Sense of oppression	2.5 (1)
Violation	5.0 (2)
Abuse	5.0 (2)
Emotional avoidance	
Risk minimization	10.0 (4)
Denial of effects	15.0 (6)
Peaceful state of mind	10.0 (4)
Anxiety for vaccine side‐effects	57.5 (23)

^a^Some participants have reported more than one theme.

## 5 DISCUSSION

The results contributed to exploring the complexities of COVID‐19 vaccination hesitancy. Specifically, the profile of “hesitant but vaccinated” seems to place between complacency (i.e., the vaccination is not a necessary action, but the decision can be influenced by many factors, such as external responsibilities) and convenience (i.e., the adherence to vaccination is given by the availability and affordability of healthcare services and procedures) postulated by the Confidence, Complacency, Convenience (3Cs) model of Vaccine Hesitancy (World Health Organization). Indeed, these participants underwent vaccinations only because this was a means of overcoming constraints. Conversely, “hesitant not vaccinated” seems to be more placed on the pole of contrariety and refusal of vaccination (“anti‐vaxxers”).

Additionally, the reasons behind the respondents’ hesitancy to vaccination fell into the three categories identified by the SAGE Working Group, namely contextual influences, individual and group influences, and vaccine/vaccine‐specific issues. Specifically, we found that the communication and media environment and the mistrust of politicians and pharmaceutical companies were relevant factors.

Indeed, we found that the mistrust of governmental authorities was considered a source of further insecurity, exacerbating the individual perception of a lack of control over one's life (Alwi et al., 2021). This finding is consistent with the scientific literature suggesting that low confidence in the government and dissatisfaction with pandemic management methods were predictive variables for refusing vaccination (Hacquin, 2020). Moreover, the recent infodemic phenomenon may negatively influence the process of vaccine acceptance as well (Germani & Biller‐Andorno, 2021). The management of contradictory information required people to exert their ability to cognitively elaborate on misleading and confusing information and to emotionally tolerate this uncertainty. A condition of uncertainty was also related to concerns and worries about possible vaccine side effects, in line with previous studies that identified fear of potential side effects as the main motivation for vaccination resistance (Alwi et al., 2021).

A further key element was the need to re‐establish control over one's life. The “hesitant not vaccinated” group re‐established it through the clear choice of not being vaccinated, which means that this group was unable to tolerate any feeling of uncertainty. Furthermore, we found conflicting emotional states: anger, a highly reactive emotion toward discrimination and social stigma, and a peaceful state of mind or indifference, working as avoidance emotional strategies to cope with fear. Notably, participants did not recognize the importance of vaccination for community protection, which is the goal of the vaccination program. Conversely, in the “hesitant but vaccinated” group, despite doubts about its effectiveness, vaccination was accepted as the only way to overcome COVID‐19 containment measures and restrictions otherwise imposed on non‐vaccinated people. These two groups differed from each other in their level of uncertainty avoidance. While “hesitant but vaccinated” individuals could somewhat tolerate a certain degree of uncertainty, it was not possible for “hesitant not vaccinated” individuals to accept such an uncertainty, probably because they could not be comfortable with ambiguity or the unknown related to possible vaccine side effects, suspending their judgment on these effects.

These findings should be interpreted in light of several limitations. First, the sample size was relatively small, and the majority of the respondents came from Northern Italy. Therefore, these results cannot be considered representative of the entire country (Alwi et al., 2021) and could be biased by the snowball sampling method. However, given that this study focused on the difficult‐to‐track and understudied “hesitant not vaccinated” population, we believe that these results significantly contribute to understanding the cognitive and emotional factors that explain vaccination hesitancy. Additionally, given its cost‐effectiveness and efficiency in reaching difficult‐to‐track participants (such as hesitant “not vaccinated” participants in our study), snowball sampling has been widely utilized by previous studies on willingness to get vaccinated against COVID‐19, especially during the pandemic, during which it was not feasible to gather data based on face‐to‐face interviews (e.g., Kong et al., 2022; Liu et al., 2021; Vizcardo et al., 2021). Moreover, our sample size was determined based on the saturation principle, which is broadly accepted in qualitative research (Saunders et al., 2018). Future studies on this topic would benefit from collecting data on larger and more nationally representative Italian samples. It is worth conducting further research to test to what extent and how uncertainty avoidance might affect an individual's willingness to be vaccinated. In doing so, research should collect data from different information sources and combine qualitative methods with quantitative ones. Secondly, we collected only a few socio‐demographic information: our sample was mainly composed of women with a variable age range and education level. However, the gender distribution in our sample is highly representative of the Italian population where men were found to be more prone to undertake vaccination (Gorman et al., 2021; Larson et al., 2016) than women who, on the contrary, seem to be more likely to show hesitant or resistant attitudes toward vaccination (Biswas et al., 2021). With regard to education, we found that in this study vaccinated individuals have higher education compared to anti‐vaxxers, in line with previous studies (Troiano & Nardi, 2021). Thirdly, in accordance with the literature, we focused our study on the main topics related to vaccine hesitance. Future research is needed to further investigate which other factors might affect an individual's willingness or unwillingness to receive the vaccine, including occupation, political orientation, and psychological factors (e.g., uncertainty avoidance, paranoia traits, and health anxiety).

## 6 CONCLUSIONS

The main psychological factor associated with vaccine hesitance in this study was the perceived lack of control and the inability to tolerate information perceived as ambiguous and uncertain (e.g., potential vaccine side effects). This uncertainty was strongly related to different emotional manifestations, such as anger, anxiety, and fear, but also emotional avoidance and indifference. It is interesting how the range of emotions that emerged can be divided into two opposing categories: on the one hand, high‐intensity negative emotions such as anger and fear, and, on the other hand, the denial of emotions manifested in a peaceful state of mind and indifference. Gaining a deeper knowledge of the key factors characterizing psychological profiles of vaccine‐hesitant/resistant individuals may provide public health officials with valuable information useful for effectively designing and delivering public health messages (Harapan et al., 2020).

With a view to primary health prevention, the findings of this study suggest that attention should be focused on the figure of the general practitioner, whose educational role can be decisive in countering and containing the overload of information caused by the vaccination. The general practitioner, thanks to the direct and trustworthy relationship with the citizen and with his family context (that was found to be so decisive in the choice of not getting vaccinated), is the most suitable figure able to accompany citizens toward effective and conscious choices for their health. Through a counseling action, in‐depth information can be conveyed, corrected, and rendered in a simple language with the possibility of activating an empowerment process that can help the citizen overcome the hesitant attitude to vaccination. These findings also highlight the importance of understanding the psychological factors that contribute to the delay in accepting or refusing COVID‐19 vaccination to maximize the positive effects of public health messages. Future qualitative studies should extend our results by further exploring in‐depth individual barriers to vaccination uptake, which primarily include psychological resistance.

## AUTHOR CONTRIBUTIONS

Conceptualization: Cecilia Perrone, Paola Gabanelli, and Elena Fiabane; Software: Marina Maffoni; Data curation: Cecilia Perrone, Paola Gabanelli, Elena Fiabane; Writing – original draft preparation: Cecilia Perrone, Paola Gabanelli; Writing – review and editing: Elena Fiabane, Marina Maffoni, Valentina Sommovigo. All authors have read and agreed to the published version of the manuscript.

## CONFLICT OF INTEREST

The authors declare no conflict of interest.

## ETHICS STATEMENT

The study was conducted in accordance with the University of Pavia and approved by the Ethics Committee of the University of Pavia (CEC N° 86/2021).

## Supporting information

Supporting Information

## Data Availability

The data that support the findings of this study are available from the corresponding author upon reasonable request.
