# Supplementary material for: Vaccination hesitancy: To be vaccinated, or not to be vaccinated, that is the question in the era of COVID‐19
Source: Public Health Nurs. 2022 Sep 27:10.1111/phn.13134. Online ahead of print. doi: 10.1111/phn.13134 (PMC9538072; doi:10.1111/phn.13134)
Supplement: Supplementary file 1 — Supporting Information [file PHN-9999-0-s001.docx]

**Supplementary Table 1**. Survey about COVID-19 vaccination hesitancy

**Socio-demographic information**

1. Age
2. Gender
3. Education
4. Residence region

**Version for Hesitant not-vaccinated subjects**

1. What are the reasons why you are not yet vaccinated?
2. If there are multiple reasons why you have not yet been vaccinated, which motivation do you feel is the main one?
3. In your life or in that of your family, have there been any previous negative health experiences?
4. What personal measures are you using to protect yourself from the virus?
5. What are the sources from which you have obtained information on vaccines? (Specify as much as possible)
6. Which of these sources had the greatest influence on the choice not to get vaccinated?
7. What are the emotions that best describe this condition of uncertainty/doubt related to vaccination?
8. Were there significant people who supported you in this still uncertain position? (e.g., People opposed to the vaccine)
9. Were there significant people who hindered you in this still uncertain position? (e.g., People favorable to the vaccine)
10. Which factors could help you to make a vaccine decision?
11. Did your indecision/choice have any particular consequence up to now?

**Version for Hesitant but vaccinated subjects**

1. What is the reason you have hesitated until today, postponing the vaccination?
2. If there are more reasons why you postponed vaccination, which is the main one?
3. In your life or in that of your family, have there been any previous negative health experiences?
4. Before you get vaccinated, what personal measures did you use to protect yourself from the virus?
5. What are the sources from which you have obtained information on vaccines? (Specify as much as possible)
6. Which of these sources had the greatest influence on the choice to postpone vaccination?
7. What were the emotions that best describe this condition of uncertainty / doubt that led you to postpone the vaccination?
8. How did you feel emotionally after the vaccination?
9. Were there significant people who supported you in this still uncertain position? (e.g., people opposed to the vaccine)
10. Were there significant people who hindered you in this still uncertain position? (e.g., people favorable to the vaccine)
11. What factors helped you decide to get vaccinated?
12. Did your indecision/choice have had any particular consequence?
